# Clinical predictors of radiographic abnormalities among infants with bronchiolitis in a paediatric emergency department

**DOI:** 10.1186/1471-2431-14-143

**Published:** 2014-06-06

**Authors:** Emmanuelle Ecochard-Dugelay, Muriel Beliah, Francis Perreaux, Jocelyne de Laveaucoupet, Jean Bouyer, Ralph Epaud, Philippe Labrune, Hubert Ducou-Lepointe, Vincent Gajdos

**Affiliations:** 1 INSERM U1018, Paris 94270, Le Kremlin Bicêtre France; 2 Paediatric Department, APHP, Hopital Antoine Béclère, BP 405, 92140 Cedex Clamart, France; 3 Department of Paediatric Radiology, APHP, Hopital Antoine Béclère, BP 405, 92140 Cedex Clamart, France; 4 Paediatric Department, Centre Intercommunal de Créteil, 94000 Créteil France; 5 University Paris Est Créteil, 94000 Créteil France; 6 Department of Paediatric Radiology, APHP, Hopital Trousseau, 75012 Paris, France; 7 University Pierre et Marie Curie, 75005 Paris, France; 8 University Paris Sud, UFR Kremlin Bicêtre, Châtenay-Malabry 94276 Cedex, Le Kremlin Bicêtre, France

**Keywords:** Bronchiolitis, Chest radiography, Radiographic pneumonia, Clinical decision rule

## Abstract

**Background:**

Acute viral respiratory exacerbation is one of the most common conditions encountered in a paediatric emergency department (PED) during winter months. We aimed at defining clinical predictors of chest radiography prescription and radiographic abnormalities, among infants with bronchiolitis in a paediatric emergency department.

**Methods:**

We conducted a prospective cohort study of children less than 2 years of age with clinical bronchiolitis, who presented for evaluation at the paediatric emergency department of an urban general hospital in France. Detailed information regarding historical features, examination findings, and management were collected. Clinical predictors of interest were explored in multivariate logistic regression models.

**Results:**

Among 410 chest radiographs blindly interpreted by two experts, 40 (9.7%) were considered as abnormal. Clinical predictors of chest radiography achievement were age (under three months), feeding difficulties, fever over 38°C, hypoxia under than 95% of oxygen saturation, respiratory distress, crackles, and bronchitis rales. Clinical predictors of radiographic abnormalities were fever and close to significance hypoxia and conjunctivitis.

**Conclusion:**

Our study provides arguments for reducing chest radiographs in infants with bronchiolitis. For infants with clinical factors such as age less than three months, feeding difficulties, respiratory distress without hypoxia, isolated crackles or bronchitis rales, careful clinical follow-up should be provided instead of chest radiography.

## Background

Acute viral respiratory exacerbation is one of the most common conditions encountered in a paediatric emergency department (PED) during winter months [1]. It is often difficult to distinguish viral from bacterial cause of dyspnea with only clinical features. The lack of reliable clinical decision rule for management of bronchiolitis often leads to perform chest radiography (CR) in order to detect radiographic abnormalities inconsistent with this diagnosis, such as pneumonia or other cardio-respiratory disease. An American survey of clinical practices in the diagnosis and management of bronchiolitis showed a CR rate of 72% that lead to an increased likelihood of antibiotic therapy and length of stay in hospital [2]. Recent studies showed that most of these CR were read as negative, (ie consistent with a simple acute viral respiratory exacerbation), and that many of them might have been avoided, saving time, money, and children exposure to ionizing radiations [3,4].

The aims of our study were to investigate historical features and clinical examination findings in the evaluation of infants with bronchiolitis that conduct to the prescription of CR, and to determine clinical predictors of radiographic abnormalities.

## Methods

### Study design and setting

The study was approved by the ethical committee CPP (Comité de Protection des Personnes) – Ile de France 3. We performed a prospective cohort study of children less than 2 years of age with clinical bronchiolitis, who presented for evaluation to the PED of a urban general hospital (Antoine Beclere – Clamart, Paris suburb, France) between October 2006 and February 2007. According to the American Academy of Pediatrics, bronchiolitis was defined as a constellation of clinical symptoms and signs including a viral upper respiratory prodrome followed by increased respiratory effort and wheezing in children less than 2 years of age [5]. All children who had clinical bronchiolitis on examination were eligible to the study. All physicians working in the PED were asked and accepted to participate. Physicians were oriented to the questionnaire before the start of the study and were continually informed of study details throughout the study period. They completed a questionnaire for each patient included. To prevent free text responses, fixed-choice format has been chosen.

### Clinical data collection

Detailed information regarding historical features and examination findings at first evaluation were collected. Demographics data included age, gender, history of prematurity, known chronic illness, and previous wheezing episodes. Historical features of interest included duration of symptoms, history of fever, feeding difficulties, and antibiotics use before PED admission. Physicians were asked to report temperature, respiratory rate, retraction signs and wheezing, oxygen saturation, auscultation findings (bronchitis rales, crackles) and the presence of otitis, conjunctivitis or toxic appearing. Clinicians recorded the following management: CR, antibiotic prescription, and decision of hospitalisation.

### Chest radiography interpretation

All CR were performed by using standard equipment and radiographic techniques. They were blindly and independently reviewed in digital format by 2 paediatric radiologists. CR was read as either normal or abnormal. Abnormalities were lobar consolidation, segmental or lobar atelectasia, or cardiomegaly. Others findings (prominent bronchial opacities, peribronchial infiltrates, hyperinflation or sub-segmental atelectasia) were considered as consistent with the diagnosis of bronchiolitis and therefore had no impact on therapeutic decision. For CR whose interpretation differed between both radiologists, we organized a consensus meeting.

### Statistical analysis

Analyses were performed on the whole study population to assess the relationship between history and clinical findings and the decision to realise CR or not. Clinical predictors of radiographic abnormality were studied on patients that underwent CR. Continuous and ordinal variables were categorized: temperature was coded as <38°C, 38 – 39°C or > 39°C, hypoxia was defined by an oxygen saturation <95%, age-related respiratory rate was defined in accordance with Liu definition (Table 1) [6]. We created a global score of respiratory distress, using weighted retraction signs widely used and that showed a good inter-observer reproducibility (Tables 2 and 3) [7]. Variables with a p-value ≤0.20 in univariate analysis were considered candidate variables for inclusion in the multivariate model and p-values ≤0.05 were considered statistically significant.

**Table 1:** **Age related respiratory rate from *Liu et al.*** [6]

	**Respiratory rate (/min)**		
	**1 point**	**2 points**	**3 points**
Age < 2 months	≤60	61-69	≥70
Age 2 – 12 months	≤50	51-59	≥60
Age 12 – 24 months	≤40	41-44	≥45

**Table 2:** Clinical score of weighted retraction signs (total /8)

	**None**	**Moderate**	**Important**
Intercostal indrawing	0	1	2
Sub-costal indrawing	0	1	2
Nasal flaring	0	1	2
Thoraco-abdominal movement	0	1	2

**Table 3:** Global score of respiratory distress

	**Global score of respiratory distress**			
	**0 point**	**1 point**	**2 points**	**3 points**
Clinical score of retraction signs	0	1 or 2	3 or 4	5 to 8

Because CR is a frequent event (about 50%), odds-ratio substantially overestimate the relative risk. We used then Poisson regression to estimate relative risk, first for performing CR, and then for radiographic abnormalities [8]. To enhance power of our analysis, we used multiple imputation for missing values [9-11].

All analyses were performed on Stata 11 Software (StataCorp. 2007. Stata Statistical Software: Release 11. College Station, TX: StataCorp LP).

## Results

### Demographics data and clinical findings

Study forms were completed for 821 patients (Table 4). One hundred–and-seventy infants (21%) were less than three months of age, 13% had prematurity history, and 60% had no wheezing history before this episode. The mean duration of symptoms was 4.1 days (SD 5.3 days), 38% of children had feeding difficulties and about 15% had antibiotic prescription before the emergency admission. Clinically, about half of children were febrile, 105 (14.1%) had oxygen saturation under 95%, 272 (33%) had bronchitis rales and 104 (12.7%) crackles. 427 (52%) of the children have had a chest radiography and 350 (42.6%) had been hospitalized. One hundred and four (12.7%) children had otitis, 27 (3.3%) conjunctivitis. 215 (26%) received antibiotics (13.5% of children without CR versus 38.8% of children with CR, p < 0.001).

**Table 4:** **Description of the study population *(n = 821)***

	**N**	**n (%)**
First wheezing episode	801	492 (59.9)
Sex (male/female)	821	476/345
Age under 3 months	821	170 (20.7)
Prematurity <37 weeks	780	108 (13.1)
Respiratory symptom length (j) – mean (SD)	806	4.1 (5.3)
Feeding difficulties	799	309 (37.6)
Temperature	738	
< 38°C		373 (45.4)
[38-39°C]		211 (25.7)
≥ 39°C		154 (18.8)
Previous antibiotherapy	816	124 (15.1)
Age-related respiratory rate [6]	747	
1 point		427 (52.0)
2 points		101 (12.3)
3 points		219 (26.7)
Global score for retraction signs	798	
0 point		233 (29.2)
1 point		454 (56.9)
2 points		83 (10.4)
3 points		28 (3.5)
Oxygen saturation	747	
≥95%		642 (85.9)
<95%		105 (14.1)
Crackles	814	81 (9.9)
Bronchitis rales	814	272 (33.1)
Otitis	789	104 (12.7)
Conjunctivitis	802	27 (3.3)
Toxic appearing	817	35 (4.3)
Chest radiography	821	427 (52.0)
Hospitalisation	821	350 (42.6)

### Chest radiographs

**Figure 1:**
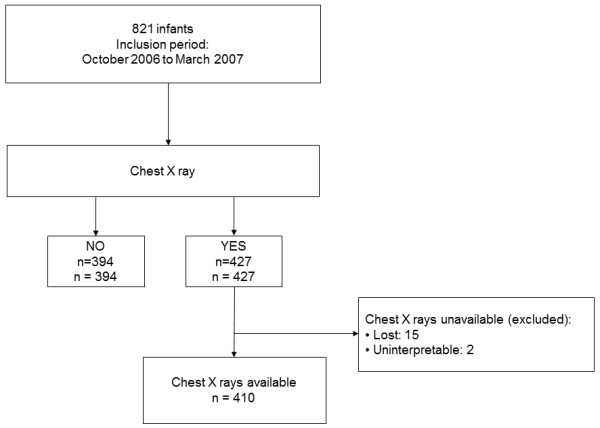
Description of chest radiographs in the study.

Among the 427 chest radiographs performed, 410 were interpreted by both experts. Two chest radiographs were excluded because of their poor quality and 15 could not be retrieved (Figure 1). Consistency rate between both blinded radiologists was 84.3%.

Most infants had prominent bronchial opacities, peribronchial infiltrates and/or hyperinflation. These CR were considered to be consistent with the diagnosis of bronchiolitis or viral acute exacerbation. Forty (9.7%) CR were considered as abnormal: 39 with lobar or alveolar condensations and one with lobar atelectasia.

### CR achievement

Univariate analysis identified nine variables that were significantly related to the realisation of a chest radiography: age ≤ 3 months (p = 0.03), feeding difficulties (p < 0.001), fever (p < 0.001), tachypnea (p = 0.002), global score of respiratory distress (p < 0.001), hypoxia (SpO2 < 95%, p < 0.001), crackles (p < 0.001), bronchitis rales (p = 0.04) and toxic appearing (p = 0.04). All these variables were included in the multivariate model (Table 5) which found a significant association between the realisation of CR and age ≤ 3 months (p = 0.003), feeding difficulties (p = 0.01), presence of fever (p < 0.001), hypoxia (p = 0.04), global score of respiratory distress (p = 0.03), crackles (p = 0.003) or bronchitis rales (p = 0.004) (Table 4).

**Table 5:** Factors associated with CR achievement: multivariate analysis

	**RR**	**IC.95**	**p**
Age ≤ 3 months	1.4	[1.1 – 1.8]	0.003
Prematurity	1.3	[1.0 – 1.6]	0.08
Feeding difficulties	1.3	[1.1 – 1.6]	0.01
Fever			
[38-39°C]	1.4	[1.1 – 1.8]	<0.001
≥ 39°C	1.9	[1.5 – 2.5]	
SpO2 < 95%	1.3	[1.0 – 1.7]	0.04
Age related respiratory rate*			
score = 2	1.2	[0.9 – 1.7]	0.2
score = 3	1.2	[1.0 – 1.5]	
Global score of respiratory distress			
score = 1	1.4	[1.1 – 1.8]	0.03
score =2	1.7	[1.2 – 2.4]	
score =3	1.7	[1.1 – 2.8]	
Toxic-appearing	1.0	[0.6 – 1.5]	0.9
Crackles	1.5	[1.2 – 2.0]	0.003
Bronchitis rales	1.4	[1.1 – 1.7]	0.004

*According to Liu et al. [6].

### Radiographic abnormalities

Univariate analysis only identified fever as significantly related to the presence of radiographic abnormality (p = 0.02). Finally, multivariate analysis only identified fever as an independent clinical predictor of radiographic abnormalities (p = 0.04) (Table 6)*.* Presence of crackles was not an independent predictor in our population (p = 0.1).

**Table 6:** Clinical predictors of radiographic abnormalities: multivariate analysis

	**RR**	**IC.95**	**p**
Fever			
[38-39°C]	1.1	[0.4 – 2.7]	0.04
≥ 39°C	2.4	[1.1 – 5.1]	
SpO2 < 95%	1.8	[0.9 – 3.5]	0.08
Conjunctivitis	2.7	[0.9 – 7.9]	0.07
History of wheezing	1.5	[0.8 – 2.8]	0.3
Crackles	1.7	[0.8 – 3.4]	0.1

## Discussion

To our knowledge, this study is one of the largest prospective cohorts of infants under two years of age who have been admitted to a PED for bronchiolitis. We have been able to determine clinical predictors of CR prescription and radiographic abnormalities. We observed a high rate of CR (52.7%) with a rate of abnormalities of 9.7% (4.9% of the whole study population). Our study found a great difference between clinical predictors of CR prescription (age ≤ 3 months, feeding difficulties, fever, presence of retraction signs, hypoxia, crackles, bronchial rales and toxic appearing) and the only clinical factor predictive of radiographic abnormalities (fever). Others clinical factors such hypoxia and conjunctivitis were not predictive of radiographic abnormalities.

The prevalence of pneumonia in previous investigations varied widely, ranging from 0.75 to 63% [3,4,12-19]*.* These variations may be due to several factors (age of patients, inclusion of patients with or without history of wheezing, percentage of children who underwent CR, considered abnormalities). Wide variations in prescription rates of CR (ranging from 42 to 72 [2,15-17]) reflect lack of consensus, even if recent guidelines recommend to limit their routine use [17]. Limiting the number of CR is important for several reasons: although the radiation associated with achieving a CR is negligible (0.02 mSv, whereas natural exposure is estimated at about 0.05 mSv per week), a recent report on the French population exposure to ionizing radiation related to acts of medical diagnosis, reports a number of CR equal to 0.2 acts per year per child under one year (approximately 160,000 procedures per year for a country with 800,000 births per year) [20]. Furthermore, CR achievement appears to be associated with an increased likelihood of prescribing unnecessary antibiotic [2,18,21]. To reduce the number of unnecessary CR, it is important to understand which clinical variables are associated with the realisation of this diagnostic test, and to compare these clinical variables with those associated with radiographic abnormalities. In our study, clinical features such as age (less than 3 months), hypoxia, conjunctivitis, feeding difficulties, retraction signs, and presence of bronchitis rales and crackles were independent clinical predictors of CR, while these variables did not appear to predict a greater risk of radiographic abnormalities. Thus, our results encourage to restrict CR prescription to infants with fever. Those clinical predictors of radiographic abnormalities are consistent with previous studies [3,4,12,13,16-18]. Neuman et al. conducted a large prospective cohort study to assess the relation between historical features and physical examination findings and radiographic pneumonia [4]. Fever, duration of fever, hypoxia and focal rales emerged as significant predictors of pneumonia on the subgroup of patients younger than five years. Furthermore they attempted to stratify children in low and high risk for pneumonia by recursive partitioning analysis but they were unable to characterize a low risk population among children of this subgroup.

As recommended, no microbiological testing has been performed in these patients. Indeed, the question of the benefit of their implementation for limiting antibiotics prescriptions or chest radiographs arises (even if a secondary bacterial infection may be a complication of an authentic viral bronchiolitis and that the presence of RSV does not exclude a bacterial superinfection).

Our study had several limitations. First it was a mono-centric study and our results could be considered representative of all PED. As CR has only been performed in 52% of infants, the scope of our assessment of factors associated with radiographic abnormalities was limited. However, the number of CR studied (410) represented a large population compared with previous studies. Moreover, given the current data in the literature and recommendations, it would be unethical to practice systematic CR in bronchiolitis. Finally, our population was heterogeneous regarding the episode of bronchiolitis considered for our study: 40% of the patients already had a wheezing episode. Our work reflected current practices in emergency room.

## Conclusion

Our study provides arguments for reducing CR achievement in infants admitted to PED for bronchiolitis. Especially, clinical factors such as age less than three months, feeding difficulties, presence of retraction signs without hypoxia, or isolated rales and crackles are not considered as predictive of radiographic abnormalities and should not lead to the prescription of CR. For those patients, careful clinical follow-up is recommended as an alternative. Moreover further studies are required to evaluate the real link between the achievement of CR and antibiotics prescription.

## Abbreviations

CR: Chest radiography; PED: Pediatric emergency department.

## Competing interests

The authors declare that they have no competing interests.

## Authors’ contributions

EDE, JB, RE, PL and VG conceived the study and its design. MB and FP coordinated inclusions in the study. JDL and HDL reviewed all chest X-rays. EDE, JB, PL and VG helped drafting the manuscript. All authors read and approved the final manuscript.

## Pre-publication history

The pre-publication history for this paper can be accessed here:

http://www.biomedcentral.com/1471-2431/14/143/prepub
